# Safety Stressors and Construction Workers' Safety Performance: The Mediating Role of Ego Depletion and Self-Efficacy

**DOI:** 10.3389/fpsyg.2021.818955

**Published:** 2022-01-17

**Authors:** Gui Ye, Qingting Xiang, Lijuan Yang, Jingjing Yang, Nini Xia, Yang Liu, Tiantian He

**Affiliations:** ^1^School of Management Science and Real Estate, Chongqing University, Chongqing, China; ^2^International Research Center for Sustainable Built Environment, Chongqing University, Chongqing, China; ^3^Department of Construction and Real Estate, School of Civil Engineering, Southeast University, Nanjing, China

**Keywords:** construction worker, safety performance, safety stressor, ego depletion, self-efficacy

## Abstract

As an important influencing factor of construction workers' safety performance, safety stressor has received increasing attention. However, no consensus has been reached on the relationship between different types of safety stressors and the subdimensions of safety performance, and the mechanism by which safety stressors influence safety performance remains unclear. This study proposed a multiple mediation model with ego depletion and self-efficacy as mediators between safety stressors and workers' safety performance. Data were collected from 335 construction workers in China. Results demonstrated that: (1) the three types of safety stressors (i.e., safety role ambiguity, safety role conflict, and interpersonal safety conflict) all had negative effects on workers' safety performance (i.e., safety compliance and safety participation); (2) self-efficacy mediated all the relationships between the three safety stressors and safety performance; (3) ego depletion only mediated part of the relationships between the three safety stressors and safety performance; and (4) only part of the multiple-step mediating effects through ego depletion and self-efficacy were supported. This study made contributions by shedding light on the mechanism by which safety stressors influence workers' safety performance and providing more empirical evidence for the relationship between various safety stressors and the subdimensions of safety performance. Additionally, targeted strategies for improving workers' safety performance were proposed according to the findings.

## Introduction

Construction is one of the most dangerous industries which incur thousands of fatal and nonfatal injuries every year (Dzeng et al., 2016; Hasanzadeh et al., 2019; Sanni-Anibire et al., 2020; Moosa and Oriet, 2021). According to the Occupational Safety and Health Administration (2019), the fatal injuries in the U.S. construction industry stood at 1008 in 2018. The corresponding figure of China was even more striking with 1752 death toll in the construction industry in the first half of 2018 (Ministry of Emergency Management of the People's Republic of China, 2018). These incidents or accidents threaten the health and safety of site personnel and bring huge losses to construction enterprises (Nodoushan et al., 2020; Zhang et al., 2020; Al-Kasasbeh et al., 2021; Zhou et al., 2021). Therefore, improving construction safety performance has always been a research hotspot. However, despite improvements over the years, safety performance in the construction industry remains unsatisfactory (Gunduz et al., 2018).

Traditionally, safety performance has been measured by lagging indicators such as the number of accidents, injury rate and death toll (Qi et al., 2022). Nevertheless, these lagging indicators may not provide the insights necessary to avoid future accidents (Grabowski et al., 2007). Hence, scholars proposed that leading indicators should be used to express safety performance (Sinelnikov et al., 2015; Shaikh et al., 2021). Leading indicators are measures of actions taken to prevent accidents (Toellner, 2001). Construction workers' performance is a typical leading indicator and has been considered as one of the ideal indicators of safety performance (Hinze et al., 2013) in that their unsafe behavior is the frequent, direct, and main cause of accidents (Jiang et al., 2015; Li et al., 2015; He et al., 2020). Eliminating workers' unsafe behaviors is the biggest challenge to improve safety performance (Fang et al., 2020), and safety performance can be achieved through workers' safety behaviors (Al-Bsheish et al., 2017). Therefore, this study defines safety performance as workers' safety behaviors. Based on the job performance theory (Borman and Motowidlo, 1993), Neal and Griffin (1997) proposed two subdimensions of safety performance, i.e., safety compliance and safety participation. Safety compliance refers to “the core safety activities that need to be carried out by individuals to maintain workplace safety,” while safety participation refers to “behaviors such as participating in voluntary safety activities or attending safety meetings” (Griffin and Neal, 2000).

The premise of improving safety performance is to understand factors that influence safety performance (Sampson et al., 2014). Occupational stressors have been widely recognized to have a significant influence on employees' job performance (Lu et al., 2016; Leung et al., 2017; Alroomi and Mohamed, 2021). Due to the complex, dynamic and uncertain site environment, construction workers have long been exposed to numerous occupational stressors (Mohr and Wolfram, 2010; Leung et al., 2016; Liang et al., 2021). Safety stressors and safety performance are occupational stressors and job performance within the safety context. Hence, the relationship between safety stressors and safety performance has attracted academic attention. Related studies have focused on examining the relationships between different safety stressors and the two subdimensions of safety performance, and evaluating the moderating effects of supervisor support, psychological capital and safety specific trust on these relationships (Sampson et al., 2014; Wang et al., 2018, 2020). In general, they believed that safety stressors had negative effects on workers' safety performance. However, the underlying mechanism by which safety stressors affect workers' safety performance remains unclear, which is not conducive to improving workers' safety performance from the perspective of safety stressors.

The relationship between occupational stressors and job performance varies according to the type of stressors and the dimension of job performance examined (Cavanaugh et al., 2000; Lepine et al., 2005; Rosen et al., 2010). In the construction industry, role ambiguity, role conflict and interpersonal conflict are the most common and representative occupational stressors (Melia and Becerril, 2007; Brockman, 2014; De Silva et al., 2017; Wu et al., 2019). Hence, this study is going to explore the effects of safety role ambiguity, safety role conflict and interpersonal safety conflict on workers' safety compliance and safety participation. Safety role ambiguity occurs when safety-related expectations and information (e.g., safety responsibilities, safety goals and safety behaviors) of workers' roles are unclear (Rizzo et al., 1970; Jackson and Schuler, 1985; Wang et al., 2020). Safety role conflict reflects that workers receive incompatible safety role expectations (Rizzo et al., 1970; Rosen et al., 2010; Akgunduz, 2015). Interpersonal safety conflict corresponds to disagreements over safety issues between organization members (Gittleman et al., 2010; Wang et al., 2020). According to the conservation of resources theory, job demands-resources model and ego depletion theory, coping with safety stressors may consume workers' resources, thus increasing workers' ego depletion and decreasing their self-efficacy. In turn, ego depletion and self-efficacy have been considered to affect safety performance (Dai et al., 2015; Adjekum, 2017). Therefore, this study explored the mechanism by which safety role ambiguity, safety role conflict and interpersonal safety conflict influence construction workers' safety compliance and safety participation from the potential mediating effects of ego depletion and self-efficacy. In addition, we also provided suggestions for the management of construction workers' unsafe behavior based on the research results.

## Theoretical Background and Hypotheses Development

### Theoretical Background

#### Conservation of Resource Theory

Conservation of resources (COR) theory was proposed by Hobfoll (1989) to explain individuals' responses to stressors. According to this theory, individuals tend to acquire, maintain, cultivate and conserve resources (Hobfoll et al., 2018). Resources refer to “anything perceived by the individual to help attain his or her goals” (Halbesleben et al., 2014, p. 1,338). Individuals use their key resources to cope with stressful situations in the current environment and they also actively construct and protect the existing resources to deal with possible stressful situations in the future. Self-control and self-efficacy are typical individual resources (Hagger et al., 2010; Guarnaccia et al., 2018; Zhong et al., 2020). Self-control refers to the self-suppression of harmful reaction tendency and self-stimulation of beneficial reaction tendency through cognitive, emotional and behavioral strategies (Hagger et al., 2010; Righetti and Finkenauer, 2011). Self-efficacy is the belief in one's ability to successfully perform a task or achieve a goal (Bandura, 1977). Addressing occupational stressors depletes individuals' resources, and the loss of resources can trigger burnout (Prapanjaroensin et al., 2017). As a result, individuals will take action to avoid resource losses (Halbesleben et al., 2014). What's more, when facing the desperate situation of resource exhaustion, the defense mechanism of individuals' self-protection will be activated and they may engage in irrational behaviors.

#### JD-R Model

Based on the COR theory, Demerouti et al. (2001) developed the job demands-resources (JD-R) model which divided job characteristics into job demands and job resources. Job demands are the requirements of work on individuals' physical, psychological, social and other aspects and the factors that require individuals to pay corresponding efforts or costs to complete the work (De Jonge and Dormann, 2006). In brief, job demands are “bad things” that consume individuals' energy at work, such as role conflict, role ambiguity and job insecurity (Bakker et al., 2005). On the contrary, job resources are “good things” at work, referring to physical, psychological, social and organizational resources that help individuals to achieve their goals, reduce job demands, and stimulate personal growth and development (Schaufeli and Bakker, 2004). There are two psychological processes that work influence employees: the stress process and the motivation process (Schaufeli, 2017). The stress process corresponds to the process that excessive job demands and lacking job resources induce burnout which in turn results in negative outcomes such as poor performance. Burnout is characterized by emotional exhaustion, depersonalization, and reduced self-efficacy (Demerouti et al., 2001). The motivation process is akin to the process that abundant job resources improve employees' work engagement and thus lead to positive effects, such as high job performance.

#### Ego Depletion Theory

Ego depletion is akin to the state of diminished self-control resulting from the depletion of self-control resources (Hagger et al., 2010). According to the ego depletion theory proposed by Baumeister et al. (1998), engaging in the act of self-control consume resources, which will impair the performance of subsequent self-control task. Ego depletion explains the failure of individuals' volitional activities such as self-control and regulation (Baumeister et al., 2007). Ego depletion occurs when individuals perform self-control actions such as coping with stress (Baumeister et al., 2007; Schmidt et al., 2007). Conversely, ego depletion will reduce employees' work engagement and output (Muraven and Baumeister, 2000; Schmeichel et al., 2003). Employees thus exhibit less organizational citizenship behavior and conduct more abnormal behaviors such as workplace deviation behavior and unsafe behavior (Dewall et al., 2007; Barnes and Wagner, 2009; Christian and Ellis, 2011; Lin et al., 2016).

### Hypotheses Development

#### The Relationship Between Safety Stressors and Safety Performance

Previous research found that hindrance job stressors had negative effects on job performance (Lepine et al., 2005; Wallace et al., 2009; Lu et al., 2016; Abbas and Raja, 2019). Safety role ambiguity, safety role conflict and interpersonal safety conflict are all hindrance job stressors that hinder workers' personal growth and goal attainment and thus these safety stressors may influence workers' safety performance (Grebner et al., 2010; Kim and Beehr, 2018). Rosen et al. (2010) proposed that the effect of stressors on performance varies with the type of stressors and the dimension of performance examined. Sampson et al. (2014) found that all safety stressors that they examined were significantly associated with decreased safety participation while only safety role ambiguity and safety role conflict were significantly related to decreased safety compliance. Based on the work of Sampson et al. (2014), Wang et al. (2018) investigated the relationship between three types of safety stressors (i.e., safety role ambiguity, safety role conflict and interpersonal safety conflict) and construction workers' safety performance. Their results indicated that the three types of safety stressors all had negative effects on construction workers' safety participation while only safety role ambiguity had significant influence on workers' safety compliance. Where after, Wang et al. (2020) examined the relationship between the three types of safety stressors and two types of safety citizenship behaviors (i.e., safety participation). Their findings suggested that safety role ambiguity, safety role conflict and interpersonal safety conflict had negative effects on proactive safety behaviors while only interpersonal safety conflict had negative effects on prosocial safety behaviors. The above studies have not reached a consensus on the relationship between the three types of safety stressors and the two subdimensions of safety performance. Therefore, this study still examined the relationship between the three types of safety stressors and the two sub-dimensions of safety performance. We hypothesize:

*H1:* Safety role ambiguity (H1a), safety role conflict (H1b) and interpersonal safety conflict (H1c) have negative effects on workers' safety compliance.
*H2*: Safety role ambiguity (H2a), safety role conflict (H2b) and interpersonal safety conflict (H2c) have negative effects on workers' safety participation.

#### The Mediating Role of Ego Depletion

##### The Relationship Between Safety Stressors and Ego Depletion

According to the ego depletion theory, engaging in self-control activities consumes the limited self-control resources, thus leading to ego depletion (Baumeister et al., 1998, 2007). A large number of studies have shown that job stressors, especially hindrance stressors (e.g., role ambiguity and role conflict), increase the depletion of individuals' self-control resources, and thus increase ego depletion (Sonnentag and Jelden, 2009; Grebner et al., 2010; Diestel and Schmidt, 2011; Prem et al., 2016; Che et al., 2017; Xia et al., 2020b). The three types of safety stressors involved in the current study are typical hindrance stressors that have negative effects on construction workers. To overcome safety stressors, workers have to use more self-control and self-regulation resources than they would under normal circumstances (Xia et al., 2020b). Therefore, safety stressors may deplete workers' self-control resources and induce ego depletion. The three types of safety stressors may deplete workers' self-control resources in different ways. First, faced with ambiguous safety roles, workers need to exert self-control to activate information-seeking and resource-seeking behaviors. Second, in the situation of safety role conflict, workers must make behavioral decisions after weighing different expectations, which calls self-control resources. Third, interpersonal safety conflict is easy to induce workers' negative emotions, such as anger, anxiety, and depression (Spector and Jex, 1998; Jiang et al., 2013; Ten Brummelhuis et al., 2014). Coping with these negative emotions requires effort in self-control (Muraven and Baumeister, 2000; Bertrams and Pahl, 2014; Prem et al., 2016). All the three types of safety stressors can increase the depletion of workers' self-control resources and thus increase workers' ego depletion. Hence, we hypothesize:

*H3*: Safety role ambiguity (H3a), safety role conflict (H3b) and interpersonal safety conflict (H3c) have positive effects on ego depletion.

##### The Relationship Between Ego Depletion and Safety Performance

Under the state of ego depletion, individuals' willingness and ability to self-control decrease, which may lead to the failure of subsequent self-control activities (Baumeister et al., 1998, 2007). Self-control is the process by which people overcome impulse, habit or automated response, and consciously control their behaviors, including inhibiting impulse to incorrect behaviors and activating correct behaviors (Tangney et al., 2004; Hagger et al., 2010; Hale and Borys, 2013). Workers' self-control is essential for maintaining a high level of safety behaviors (Probst and Brubaker, 2001). Failures of self-control lead to an increase in risky behaviors or unsafe behaviors, thereby impairing safety performance (Salmon et al., 2014; Dai et al., 2015). For example, minor violations are the prepotent response of workers and may be reinforced into habitual violations that are carried out in a non-thinking and automated way (Reason et al., 1998; Hinsz et al., 2007). As workers' self-control ability and willingness decline, their resistance to the impulse of automated behaviors also declines. Therefore, they cannot resist the habitual impulse to violate safety regulations, resulting in increased violations and reduced safety compliance. In addition, according to the COR theory (Hobfoll, 1989), workers are inclined to conserve their limited resources. Especially when they have consumed self-control resources, they would be more cautious about subsequent resource allocation. Trougakos et al. (2015) proposed that employees would devote their resources to fulfilling work tasks rather than extra-role organizational citizenship behaviors. Similarly, workers who experience ego depletion would devote less effort toward organizational citizenship behaviors, thus reducing safety participation. Accordingly, we hypothesize:

*H4*: Ego depletion has negative effects on safety compliance (H4a) and safety participation (H4b).

According to the COR theory and JD-R model, stress factors or job demands such as safety role ambiguity, safety role conflict and interpersonal safety conflict consume workers' resources. The ego depletion theory further suggests that workers need to invest more self-control resources when experiencing safety role ambiguity, safety role conflict and interpersonal safety conflict. The depletion of self-control resources puts workers into the state of ego depletion (Hagger et al., 2010). Workers' ability and willingness to engage in subsequent self-control activities decrease. Accordingly, workers may engage in more violations (e.g., unsafe behavior) or perform less organizational citizenship behavior (Barnes and Wagner, 2009; Lin et al., 2016). In brief, dealing with safety role ambiguity, safety role conflict and interpersonal safety conflict increase the possibility of workers' ego depletion which may damage safety performance. That is, ego depletion may mediate the relationship between safety stressors and safety performance. As a result, we hypothesize:

*H5*: Ego depletion mediates the relationships between safety stressors [safety role ambiguity (H5a), safety role conflict (H5b) and interpersonal safety conflict (H5c)] and safety compliance.
*H6*: Ego depletion mediates the relationships between safety stressors [safety role ambiguity (H6a), safety role conflict (H6b), interpersonal safety conflict (H6c)] and safety participation.

#### The Mediating Role of Self-Efficacy

##### The Relationship Between Safety Stressors and Self-Efficacy

The JD-R theory proposes that high job demands may lead to burnout (Demerouti et al., 2001). Reduced self-efficacy is the core characteristic of burnout (Maslach, 1982). Namely, job demands can lead to a decrease in self-efficacy. Role ambiguity, role conflict and interpersonal conflict are widely regarded as a kind of hindering job demands (Lorente Prieto et al., 2008; Ashill and Rod, 2011; Martinez-Corts et al., 2015; Kilroy et al., 2016; Kim and Beehr, 2018; Kim et al., 2020). Previous studies have also shown that role ambiguity, role conflict and interpersonal conflict were negatively correlated with individuals' self-efficacy (Jex and Gudanowski, 1992; Hartline and Ferrell, 1996; Chebat and Kollias, 2000; Eys and Carron, 2001; Karatepe et al., 2006; Li and Bagger, 2008; Tang and Chang, 2010; Kadir et al., 2017). We can infer that safety role ambiguity, safety role conflict and interpersonal safety conflict may contribute to the decrease of workers' self-efficacy. To be exact, safety role ambiguity means that workers lack sufficient information to properly assess their ability to perform safety tasks, thus inhibiting their ability to visualize their performance. This reduces workers' confidence in their ability to complete safety tasks (Bandura, 1977). Experiencing safety role conflict and interpersonal safety conflict can also reduce workers' self-efficacy because conflicting environment makes workers question their ability (Tang and Chang, 2010).

*H7*: Safety role ambiguity (H7a), safety role conflict (H7b) and interpersonal safety conflict (H7c) have negative effects on self-efficacy.

##### The Relationship Between Self-Efficacy and Safety Performance

Self-efficacy has been proposed to significantly and positively correlate with safety performance (Chen and Chen, 2014; Adjekum, 2017; Kim and Jung, 2019). Workers with a high level of self-efficacy show more initiative at work and are more willing to learn new skills, making more efforts to understand safety procedures as well as learning skills that are necessary for them to do their work safely (Chughtai, 2015), which may increase their awareness and ability to perform safety compliance. Likewise, workers who have more belief in self-efficacy are more confident in their ability to complete extra-role tasks (Parker, 2000). They are more likely to participate in safety activities and help colleagues, thus increasing safety participation. On the contrary, workers with low self-efficacy have less confidence in their ability to complete safety-related tasks. They do not trust that they could be more professional than others. Therefore, they are not inclined to voice their safety opinions and help colleagues with safety issues. Nor do they think they can learn more safety skills or understand more safety rules. As a result, workers who have less belief in self-efficacy may have poor safety performance.

*H8*: Self-efficacy has positive effects on safety compliance (H8a) and safety participation (H8b)

As safety stressors can reduce workers' self-efficacy and workers' self-efficacy positively correlate with their safety performance, we can infer that self-efficacy may mediate the relationship between safety stressors and safety performance. Thus, we hypothesize:

*H9*: Self-efficacy mediates the relationships between the three safety stressors [safety role ambiguity (H9a), safety role conflict (H9b), interpersonal safety conflict (H9c)] and safety compliance.
*H10*: Self-efficacy mediates the relationships between the three safety stressors [safety role ambiguity (H10a), safety role conflict (H10b), interpersonal safety conflict (H10c)] and safety participation.

#### The Multiple-Step Mediating Effects Through Ego Depletion and Self-Efficacy

Previous studies show that self-efficacy is also affected by ego depletion (Chow et al., 2015; Graham and Steven, 2015). Self-efficacy is not an entirely automated process but one that requires self-control, since individuals need to ignore or deal with doubt and fear to maintain confidence (DeBono and Muraven, 2013). Workers who experience ego depletion may find it necessary to conserve resources (Job et al., 2010). They would reduce their self-efficacy for subsequent self-control to conserve resources (Chow et al., 2015). Furthermore, workers who suffer ego depletion may have more negative evaluations of themselves and more negative predictions of their subsequent performance (DeBono and Muraven, 2013). In other words, ego depletion makes workers believe that they are inefficacious (Chow et al., 2015), thus decreasing their self-efficacy in subsequent tasks (Fischer et al., 2007; Graham and Steven, 2015; Graham et al., 2017).

*H11*: Ego depletion has negative effects on self-efficacy.

As stated above, the three types of safety stressors may have positive effects on workers' ego depletion. Ego depletion influences workers' self-efficacy which, in turn, impacts their safety performance (Adjekum, 2017). Therefore, there may exist multiple-step mediation effects of safety stressors on safety performance through ego depletion and then self-efficacy. In other words, safety stressors induce workers' ego depletion which decreases their self-efficacy, thus reducing their safety performance.

*H12*: Safety role ambiguity (H12a), safety role conflict (H12b), and interpersonal safety conflict (H12c) impair construction workers' safety compliance through the multiple-step mediating effect of ego depletion and self-efficacy.
*H13*: Safety role ambiguity (H13a), safety role conflict (H13b), and interpersonal safety conflict (H13c) impair construction workers' safety participation through the multiple-step mediating effect of ego depletion and self-efficacy.

**Figure 1:**
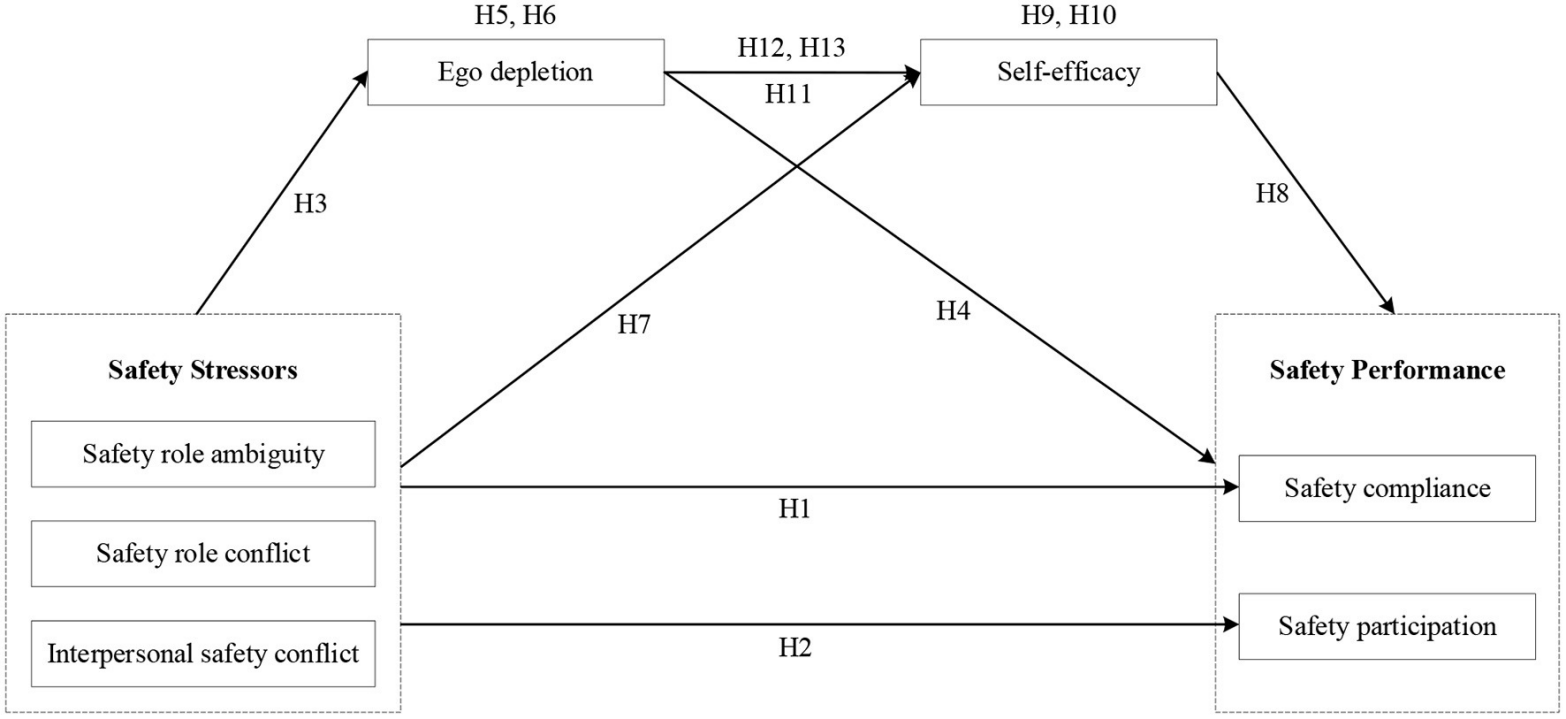
The conceptual model.

The conceptual model that integrates all hypotheses stated above is shown in Figure 1.

## Methods

### Participants and Data Collection Procedures

A questionnaire survey was conducted to collect data for hypothesis tests. Before the formal survey, a pre-research was conducted in a construction project in Chengdu, China. 15 construction workers from this project participated in the pre-research. The preliminary questionnaire was slightly modified to make it easier to understand based on the feedback from the 15 construction workers. The formal survey was carried out at seven construction sites in Chongqing, China, from October 2018 to March 2019. A total of 400 questionnaires were sent out and taken back on the spot. Questionnaires with more than 5% unanswered items [*N* = 40; according to Seo (2005) and Xia et al. (2020a)] and those answered arbitrarily (*N* = 25; e.g., there is an obvious pattern of repetition in the answers) were manually identified and excluded. Therefore, the final valid sample size was 335 (83.75% valid response rate).

Respondents' demographic characteristics are shown in Table 1. As for gender distribution, most respondents were male, accounting for 87.5% (*n* = 293), while female respondents accounted for only 12.5% (*n* = 42). All respondents were over the age of 20 and those aged 41–50 made up the largest proportion (45.70%), almost half of the total. 78% (*n* = 263) of the surveyed workers had been working in the construction industry for more than 5 years. In terms of educational background, most participants were poorly educated, with 73.1% (*n* = 245) of respondents completing only primary or secondary school.

**Table 1:** Demographic characteristics of the respondents (*N* = 335).

**Characteristics**	**Items**	**Frequency**	**Percentage (%)**
Gender	Male	293	87.5
	Female	42	12.5
Age	20–30	73	21.80
	31–40	77	23.00
	41–50	153	45.70
	51–60	31	9.30
	More than 60	1	0.30
Work experience	<5 years	72	21.50
	5–10 years	119	35.50
	11–15 years	65	19.40
	16–20 years	54	16.10
	21–25 years	21	6.30
	26–30 years	4	1.20
Educational background	Primary school or below	107	31.90
	Secondary school	138	41.20
	High school	35	10.40
	Junior college	28	8.40
	Undergraduate or above	27	8.10

### Measures

The constructs of this study consist of safety stressors, ego depletion, self-efficacy, and safety performance. The scales for measuring these variables were adopted and modified from previous studies. All measurement items were rated with a five-point Likert scale ranging from 1 to 5.

#### Safety Stressors

Consistent with the research of Sampson et al. (2014), Wang et al. (2018), and Wang et al. (2020), 18 items were adopted and modified to measure the three types of safety stressors, i.e., safety role ambiguity (SRA, five items), safety role conflict (SRC, nine items), and interpersonal safety conflict (ISC, four items). Sample items of the three sub-scales included “There are not clear, planned safety goals and objectives for my job,” “I have to ignore a rule or policy to carry out an assignment safely,” and “I get into arguments about safety with others at work,” respectively. The scales of safety role ambiguity and safety role conflict were rated based on the level of agreement ranging from 1 (strongly disagree) to 5 (strongly agree), while the scale of interpersonal safety conflict was rated based on the frequency of occurrence varying from 1 (never) to 5 (extremely often).

#### Ego Depletion

The measurement scale of ego depletion (ED) was adopted and modified from Johnson et al. (2014). 10 items were used to measure construction workers' ego depletion, including items like “I feel drained.” Items of the ego depletion scale were rated ranging from 1 (strongly disagree) to 5 (strongly agree).

#### Self-Efficacy

The measurement of self-efficacy (SE) was referred to the general self-efficacy scale developed by Schwarzer et al. (1997). A sample item of the 10-items scale is “I can always manage to solve difficult problem if I try hard enough.” Participants were asked to score each item using the number of 1 (strongly disagree) to 5 (strongly agree).

#### Safety Performance

The measurement scale of safety performance used in this study was developed by Griffin and Hu (2013), with four items measuring safety compliance (SC) and four items measuring safety participation (SP). Measuring items included “I use the correct safety procedures for carrying out my job,” “I help my coworkers when they are working under risky or hazardous conditions,” and so on. All items were rated from 1 (strongly disagree) to 5 (strongly agree).

### Data Analysis Procedures

First, SPSS 22.0 was used for descriptive statistical analysis of the questionnaire data, through which the mean, standard deviation (SD) and correlation coefficients of the variables were obtained. Second, reliability analysis and validity analysis were employed to evaluate the quality of the measurement model. Reliability was assessed by Cronbach's alpha value of variables. Validity analysis included convergent validity test and discriminant validity test. Then, a structural equation model was constructed to test the research hypotheses. In line with Baron and Kenny (1986), a three-step method was applied to examine the condition for establishing mediation. The first step was to examine the effect of independent variables on dependent variables (testing H1 and H2). In the second step, the influence of independent variables on meditators and the effect of mediators on dependent variables were examined (testing H3, H4, H7, and H8). The last step was to develop a structural equation model of the multiple mediation model to examine the mediation effects (testing H5, H6, H12, and H13). Bias-corrected (BC) bootstrap method was used to define the confidence intervals (CI) for examining the significance of the indirect effects.

## Results

### Descriptive Statistics

The mean and standard deviation (SD) of variables, and the correlation coefficients among variables were shown in Table 2. Safety role ambiguity, safety role conflict, and interpersonal safety conflict were negatively related to safety compliance and safety participation. Safety role ambiguity, safety role conflict and interpersonal safety conflict were positively related to ego depletion. Safety role ambiguity, safety role conflict and interpersonal safety conflict were negatively related to self-efficacy. Ego depletion was negatively related to self-efficacy, safety compliance and safety participation. Self-efficacy was positively related to safety compliance and safety participation.

**Table 2:** Means, SD, and correlation coefficients among variables.

**Variables**	**Mean**	**SD**	**1**	**2**	**3**	**4**	**5**	**6**	**7**
1. SRA	3.77	0.85	**0.783**						
2. SRC	3.68	0.85	0.458*	**0.693**					
3. ISC	2.29	0.85	0.211**	0.209**	**0.712**				
4. ED	3.43	0.86	0.205**	0.341**	0.396**	**0.709**			
5. SE	3.34	0.84	−0.459**	−0.493**	−0.435**	−0.364**	**0.673**		
6. SC	4.10	0.99	−0.571**	−0.410**	−0.427**	−0.192*	0.457**	**0.851**	
7. SP	3.73	1.02	−0.536**	−0.322*	−0.397**	−0.184**	0.345**	0.552**	**0.785**

* *p < 0.05*,

** *p < 0.01*.

*Diagonal bold font indicates the square root of AVE. The lower triangle presents Pearson's correlation coefficients between variables*.

### Reliability and Validity Testing

The quality of the measurement model was assessed by reliability and validity testing. Reliability was tested with Cronbach's alpha value. As shown in Table 3, Cronbach's alpha value of variables ranged from 0.799 to 0.912, reaching the accepted threshold value of 0.7 (Nunnally, 1978). Hence, it can be concluded that the measurement model had good reliability. Both convergent validity and discriminant validity were tested in this study. Convergent validity was assessed by the indices of standard factor loading (SFL), construct reliability (CR), and average variance extracted (AVE). Results of the convergent validity testing are presented in Table 3. To ensure good convergent validity, the SFL values should exceed 0.5 (Hair et al., 2006), while the CR values and AVE values should be >0.6 and 0.5, respectively (Fornell and Larcker, 1981). It can be seen from Table 3 that SFL were all significant (*p* < 0.001) and most indicators were above 0.5 (only one indicator being less than 0.5). CR values ranged from 0.804 to 0.913. And most of the AVE values reached the threshold of 0.5. Therefore, the convergence reliability of the measurement model was acceptable. Discriminant validity was examined by comparing the square root of AVE and the correlation coefficients between variables. The square root of a variable's AVE should be higher than the correlation coefficients involving that variable (Fornell and Larcker, 1981). The Pearson's correlation coefficients between variables and the square root of AVE are shown in Table 2. All the square root of AVE was higher than the involving correlation coefficients, revealing that the discriminant validity of each construct was acceptable.

**Table 3:** Results of reliability and validity testing.

**Variables**	**Indicators**	**SFL**	**S.E**.	**Est./S.E**.	***p*** **-value**	**Cronbach's alpha**	**CR**	**AVE**
SRA	SRA1	0.819	0.023	36.179	***	0.886	0.888	0.613
	SRA2	0.706	0.031	22.517	***			
	SRA3	0.801	0.024	33.234	***			
	SRA4	0.846	0.021	40.948	***			
	SRA5	0.735	0.029	25.335	***			
SRC	SRC1	0.627	0.037	16.982	***	0.891	0.892	0.480
	SRC2	0.731	0.029	24.980	***			
	SRC3	0.592	0.039	15.195	***			
	SRC4	0.707	0.031	22.787	***			
	SRC5	0.737	0.029	25.489	***			
	SRC6	0.672	0.034	19.929	*			
	SRC7	0.731	0.029	24.965	***			
	SRC8	0.715	0.031	23.494	***			
	SRC9	0.708	0.031	22.819	***			
ISC	ISC1	0.616	0.042	14.734	***	0.799	0.804	0.507
	ISC2	0.742	0.035	21.508	***			
	ISC3	0.752	0.034	22.217	***			
	ISC4	0.731	0.036	20.540	***			
ED	ED1	0.698	0.031	22.703	***	0.5	0.908	0.502
	ED2	0.753	0.026	28.477	***			
	ED3	0.808	0.022	36.679	***			
	ED4	0.681	0.032	21.304	***			
	ED5	0.422	0.047	8.917	***			
	ED6	0.726	0.029	25.411	***			
	ED7	0.744	0.027	27.370	***			
	ED8	0.612	0.037	16.597	***			
	ED9	0.829	0.020	40.506	***			
	ED10	0.727	0.028	25.525	***			
SE	SE1	0.821	0.022	37.338	***	0.86	0.891	0.453
	SE2	0.715	0.030	23.725	***			
	SE3	0.591	0.039	15.231	***			
	SE4	0.698	0.031	22.234	***			
	SE5	0.707	0.031	22.960	***			
	SE6	0.625	0.037	17.077	***			
	SE7	0.672	0.033	20.120	***			
	SE8	0.511	0.044	11.698	***			
	SE9	0.721	0.030	24.254	***			
	SE10	0.622	0.037	16.940	***			
SC	SC1	0.797	0.023	34.481	***	0.92	0.913	0.725
	SC2	0.852	0.019	45.537	***			
	SC3	0.876	0.017	52.282	***			
	SC4	0.878	0.017	52.360	***			
SP	SP1	0.676	0.034	19.742	***	0.863	0.865	0.617
	SP2	0.826	0.024	34.340	***			
	SP3	0.798	0.026	31.210	***			
	SP4	0.831	0.024	34.915	***			

* *p < 0.05*,

*** *p < 0 .001*.

### Hypothesis Testing

Structural equation modeling (SEM) was employed in the present study to test hypotheses since SEM is very effective in controlling measurement errors when estimating both the direct and indirect effects (Cheung and Lau, 2008). According to the suggestion of Baron and Kenny (1986), a causal steps strategy was used to examine the condition for establishing mediation.

**Figure 2:**
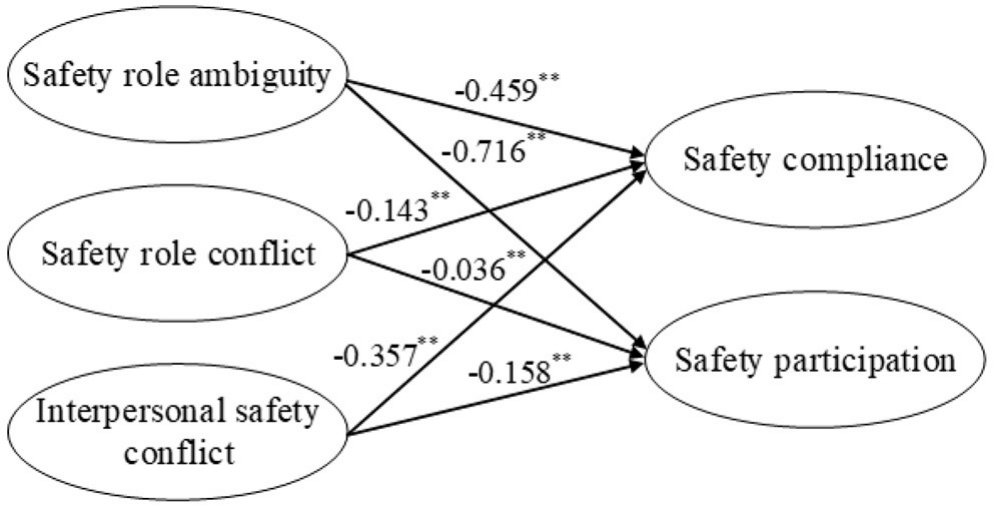
Direct effects of safety stressors on safety performance. **p < 0.01.

First, the direct effects of the independent variables on the dependent variables were examined. The path coefficients among safety stressors and safety performance are shown in Figure 2. Safety role ambiguity, safety role conflict, and interpersonal safety conflict negatively influenced safety compliance, which supported H1a, H1b, and H1c. Safety role ambiguity, safety role conflict, and interpersonal safety conflict had negative effects on safety participation, thus supporting H2a, H2b, and H2c. All the independent variables were found to have significant effects on the dependent variables. Therefore, the first condition for establishing mediation was supported.

**Figure 3:**
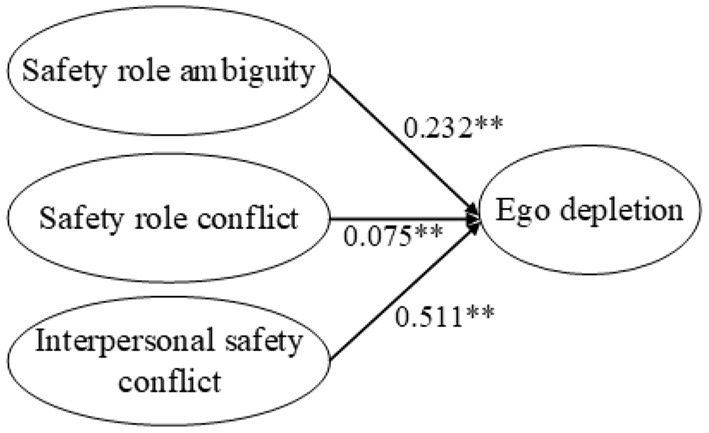
Direct effects of safety stressors on ego depletion. **p < 0.01.

**Figure 4:**
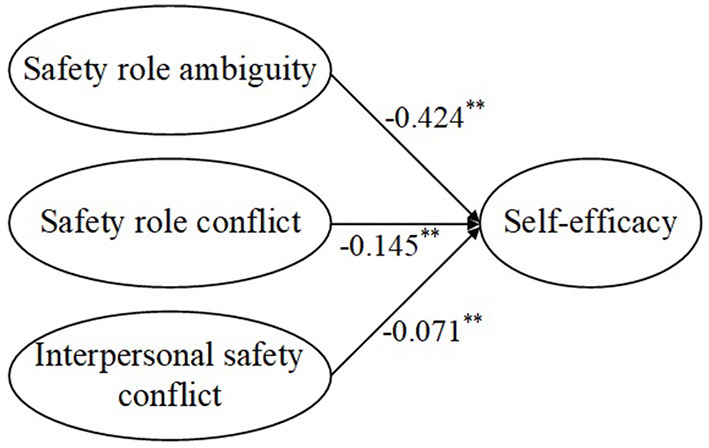
Direct effects of safety stressors on self-efficacy. **p < 0.01.

**Figure 5:**
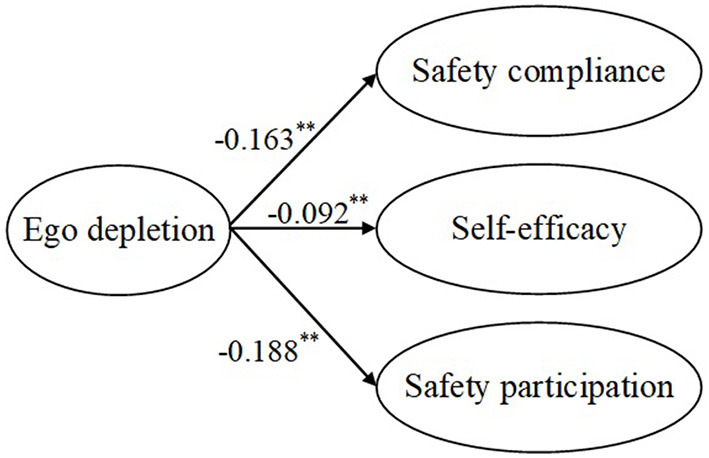
Direct effects of ego depletion on safety performance and self-efficacy. **p < 0.01.

**Figure 6:**
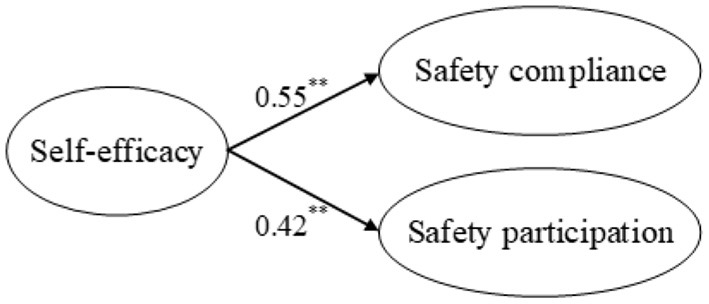
Direct effects of self-efficacy on safety performance. **p < 0.01.

Second, the direct effects of the independent variables on the mediators and the effect of the mediators on the dependent variables were examined. Figure 3 shows the path coefficients among safety stressors and ego depletion, and Figure 4 shows the path coefficients among safety stressors and self-efficacy. Safety role ambiguity, safety role conflict, and interpersonal safety conflict positively affected ego depletion, supporting H3a, H3b, and H3c. Safety role ambiguity, safety role conflict, and interpersonal safety conflict had negative effects on self-efficacy, which supported H7a, H7b, and H7c. Figure 5 presents the path coefficients among ego depletion, self-efficacy, and safety performance, and Figure 6 presents the path coefficients among self-efficacy and safety performance. Ego depletion negatively influenced safety compliance and safety participation, which meant that H4a and H4b were supported. Self-efficacy had positive effects on safety compliance and safety participation, thus supporting H8a and H8b. Ego depletion was found to negatively affect self-efficacy, which supported H11. All independent variables had significant effects on the mediators and the mediators had significant effects on the dependent variables. Hence, the second condition of mediation was also supported, suggesting that ego depletion and self-efficacy may act as mediators in the relationship between safety stressors and safety performance.

**Figure 7:**
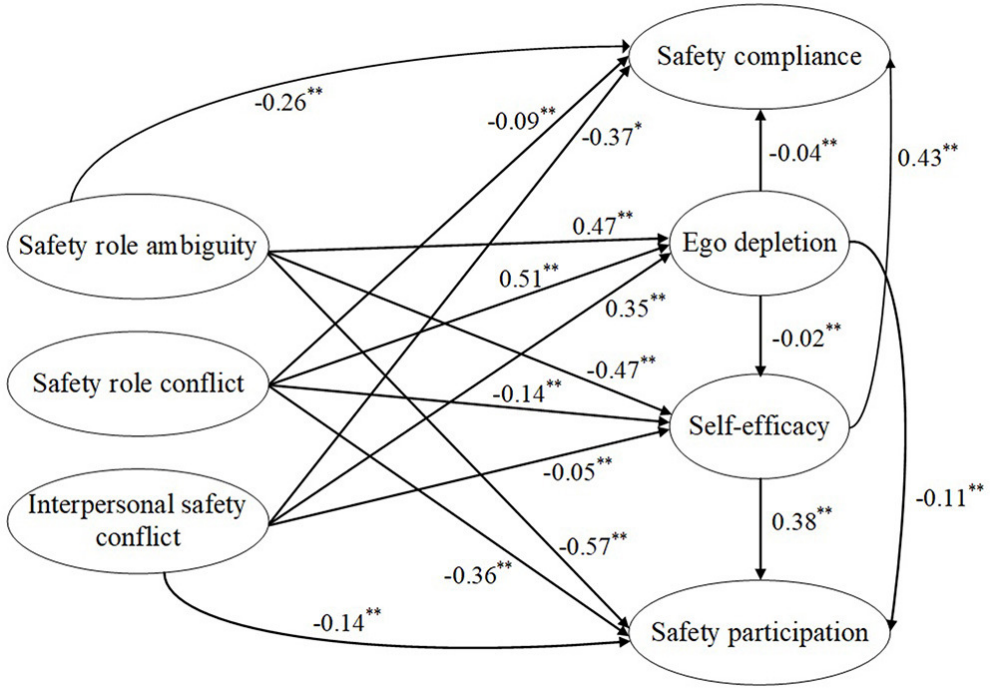
Structural equation model of the conceptual model. *p < 0.05; **p < 0.01 (two-tailed); *N* = 335.

A structural equation model of the multiple mediation model was developed to test the mediation effects, and the results are shown in Figure 7. The overall fit index (χ^2^ = 1,317.14, *df* = 968, χ^2^ / *df* = 1.36, CFI = 0.952, TLI = 0.949, RMSEA = 0.033, SRMR = 0.045) indicated that the overall fitness of the structural model was good. Following the suggestion of Cheung and Lau (2008), we used the bias-corrected (BC) bootstrap method to define the confidence intervals (CI) for examining the significance of the indirect effects. The bootstrap sample size and the confidence intervals were set as 1,000 and 95%, respectively. Table 4 shows the standardized direct effects, indirect effects, and total effects of the hypothesized mediation model. Interpersonal safety conflict had significant indirect effects on safety compliance through ego depletion, which meant that ego depletion mediated the relationship between interpersonal safety conflict and safety compliance (supporting H5c). Safety role conflict had significant indirect effects on safety participation through ego depletion, suggesting that ego depletion mediated the relationship between safety role conflict and safety participation (supporting H6b). The indirect effect of interpersonal safety conflict on safety compliance through ego depletion was significant, supporting H6c (i.e., “ego depletion mediated the relationship between interpersonal safety conflict and safety compliance”). Safety role ambiguity influenced safety compliance through self-efficacy, thus supporting H9a. Safety role conflict also affected safety compliance through self-efficacy, thus supporting H9b. Interpersonal safety conflict had a significant indirect effect on safety compliance through self-efficacy, which meant that self-efficacy mediated the relationship between interpersonal safety conflict and safety compliance (supporting H9c). Safety role ambiguity had significant indirect effects on safety participation through self-efficacy, which suggested that self-efficacy mediated the relationship between safety role ambiguity and safety participation (supporting H10a). The indirect effect of safety role conflict on safety participation through self-efficacy was significant, supporting H10b (i.e., “self-efficacy mediated the relationship between safety role conflict and safety participation”). The indirect effect of interpersonal safety conflict on safety participation through self-efficacy was significant, supporting H10c (i.e., “self-efficacy mediated the relationship between interpersonal safety conflict and safety participation”). H12c (i.e., “interpersonal safety conflict impaired construction workers' safety compliance through the multiple mediating effects of ego depletion and self-efficacy”) was supported as interpersonal safety conflict had significant indirect effects of on safety compliance through ego depletion and self-efficacy. Additionally, H13b (i.e., “safety role conflict impaired construction workers' safety participation through the multiple mediating effects of ego depletion and self-efficacy”) was supported since the indirect effect of safety role conflict on safety participation through ego depletion and self-efficacy was significant. However, the rest of the estimated indirect effects were insignificant (*p* > 0.05), thus rejecting H5a, H5b, H6a, H12a, H12b, H13a, and H13c. In addition, the direct effects of the three safety stressors on safety compliance and safety participation were significant (see Table 4), suggesting that ego depletion and self-efficacy only partially mediated the relationship between safety stressors and safety performance.

**Table 4:** Standardized direct effects, indirect effects, and total effects of the conceptual model.

	**Estimate**	**S.E**.	***P*** **-value**	**95% bias-corrected CI**	
				**Lower**	**Upper**
**Standardized direct effects**					
SRA—SC	−0.26	0.03	**	0.01	0.51
SRC—SC	−0.09	0.08	**	0.06	0.24
ISC—SC	−0.37	0.02	*	0.61	0.14
SRA—SP	−0.57	0.05	**	0.29	0.87
SRC—SP	−0.36	0.01	**	0.23	0.17
ISC—SP	−0.14	0.03	**	0.10	0.39
**Standardized indirect effects**					
SRA—ED—SC	−0.02	0.05	0.32	−0.06	0.13
SRA—SE—SC	−0.20	0.10	**	0.04	0.44
SRA—ED—SE—SC	−0.26	0.02	0.19	−0.15	0.32
SRA—ED—SP	−0.05	0.05	0.43	−0.04	0.17
SRA—SE—SP	−0.18	0.06	**	0.03	0.39
SRA—ED—SE—SP	0.00	0.02	0.22	−0.05	0.02
SRC—ED—SC	−0.02	0.05	0.23	−0.12	0.08
SRC—SE—SC	−0.06	0.04	**	0.03	0.17
SRC—ED—SE—SC	−0.05	0.02	0.29	−0.02	0.06
SRC—ED—SP	−0.13	0.06	**	0.07	0.15
SRC—SE—SP	−0.11	0.04	*	0.04	0.16
SRC—ED—SE—SP	−0.08	0.02	**	0.05	0.13
ISC—ED—SC	−0.05	0.04	**	0.03	0.09
ISC—SE—SC	−0.08	0.05	**	0.06	0.13
ISC—ED—SE—SC	−0.03	0.01	**	0.02	0.05
ISC—ED—SP	−0.19	0.05	*	0.16	0.22
ISC—SE—SP	−0.09	0.04	**	0.05	0.12
ISC—ED—SE—SP	−0.07	0.01	0.19	0.01	0.09
**Standardized total effects**					
SRA—SC	−0.74	0.02	**	0.32	0.88
SRC—SC	−0.18	0.02	**	0.03	0.25
ISC—SC	−0.53	0.01	**	0.16	0.59
SRA—SP	−0.80	0.02	**	0.58	1.01
SRC—SP	−0.68	0.01	**	0.14	0.72
ISC—SP	−0.49	0.01	**	0.09	0.54

* *p < 0.05*,

** *p < 0.01*.

## Discussion

Prior studies have discussed the effects of safety role ambiguity, safety role conflict, and interpersonal safety conflict on workers' safety performance, but they did not agree on the relationship between these safety stressors and safety performance. They have examined the moderating effects of supervisor support, psychological capital, and safety specific trust on these relationships, but they did not explore the mediating variables in these relationships. Given this, this study investigated the relationships between the three safety stressors and construction workers' safety performance. Moreover, this study also examined the mediating role of ego depletion and self-efficacy in the relationships between the three safety stressors and construction workers' safety performance.

### Theoretical Implications

First, safety role ambiguity, safety role conflict and interpersonal safety conflict had negative effects on both safety compliance and safety participation. This finding was not completely consistent with previous studies. Sampson et al. (2014) and Wang et al. (2018) also found that safety role ambiguity, safety role conflict and interpersonal safety conflict negatively influenced construction workers' safety participation. However, only parts of the relationships between the three safety stressors and safety compliance were supported in their studies. The discrepancy between our results and the results of Sampson et al. (2014) may be attributed to the different subjects we surveyed. The participants of Sampson et al. (2014) were pipefitters from the United States while our respondents were construction workers from China. Both job functions and cultural differences may have influenced the results. As for the inconsistent results between this study and the study of Wang et al., it may be because we used different safety performance measurement scales. Wang et al. (2018) applied the scale developed by Griffin and Neal (2000). We employed the scale modified by Griffin and Hu (2013). Although this finding is not consistent with the research results of Sampson et al. (2014) and Wang et al. (2018), it is congruent with the general job stressors-performance research (Jex, 1998; Wallace et al., 2009; Rosen et al., 2010; Eatough et al., 2011). Safety role ambiguity, safety role conflict and interpersonal safety conflict were hindrance stressors that could reduce job performance (Lepine et al., 2005; Abbas and Raja, 2019). Safety role ambiguity created uncertainty for workers and weakened their motivation to maintain and improve safety performance (Beehr and Bhagat, 1985; Celik, 2013). Safety role conflict reflected that workers receive incompatible safety role expectations (Sampson et al., 2014). The most common example is that the organization requires workers to both comply with safety rules and work faster, but sometimes the two goals conflict. Abiding by safety rules is often time-consuming and requires extra efforts, which may affect production and annoy colleagues. To avoid criticism, workers tend to eschew such safe behaviors (Fang et al., 2016), which may decrease safety performance. Interpersonal safety conflict could bring negative emotions (Barki and Hartwick, 2004) which may lead to workers' deliberate violations, thereby reducing safety compliance. When conflicts arise between workers and their colleagues, they tend to avoid them and be reluctant to help them with safety issues (Curcuruto et al., 2019), namely, hindering their safety participation.

Second, ego depletion mediated parts of the examined relationships between safety stressors and safety performance. Safety role ambiguity, safety role conflict, and interpersonal safety conflict had significant positive effects on workers' ego depletion which meant that coping with these stressors consumed workers' self-control resources. Ego depletion, in turn, had negative effects on workers' safety compliance and safety participation. The ego depletion theory and COR theory provided explanations for this phenomenon. According to the ego depletion theory, ego depletion leads to the decrease of an individual's willingness and ability to subsequent self-control, resulting in fewer organizational citizenship behaviors and more unsafe behaviors (Baumeister et al., 1998, 2007; Dai et al., 2015). The COR theory suggests that individuals will be more cautious about resource allocation when they lack resources. Faced with heavy tasks and multiple work objectives, construction workers may allocate their limited self-control resources to more important tasks rather than in safety activities, resulting in reduced safety performance (Xia et al., 2019). Safety role ambiguity, safety role conflict and interpersonal safety conflict had significant effects on workers' ego depletion and ego depletion had significant effects on safety compliance and safety participation. However, ego depletion was found to only mediate the relationship between interpersonal safety conflict and safety compliance, the relationship between safety role conflict and safety participation, as well as the relationship between interpersonal safety conflict and safety participation.

Third, self-efficacy mediated all the examined relationships between safety stressors and safety performance. Safety role ambiguity, safety role conflict, and interpersonal safety conflict had negative effects on workers' self-efficacy. This finding could be explained by the JD-R model. Safety role ambiguity, safety role conflict, and interpersonal safety conflict were job demands (Bakker et al., 2005). High job demands could induce burnout and reduced self-efficacy was the core characteristic of burnout (Demerouti et al., 2001). Namely, these safety stressors negatively affected workers' self-efficacy. Self-efficacy was found to have positive effects on workers' safety compliance and safety participation, which was consistent with previous studies (Chen and Chen, 2014; Adjekum, 2017; Kim and Jung, 2019). Self-efficacious workers had better feelings of work control and motivation to work safely, which may foster their safety behaviors (He et al., 2019). Safety role ambiguity, safety role conflict, and interpersonal safety conflict had significant effects on workers' self-efficacy and workers' self-efficacy had significant effects on workers' safety compliance and safety participation. Thus, we proposed that self-efficacy may mediate the relationships between safety stressors and safety performance. The results of the empirical study supported our hypothesis. The mediating effects of self-efficacy on the relationship between the three types of safety stressors and the two sub-dimensions of safety performance were all statistically significant. That is, safety role ambiguity, safety role conflict and interpersonal safety conflict could influence workers' safety performance through workers' self-efficacy.

In addition, ego depletion was found to have negative effects on workers' self-efficacy. This was because maintaining self-efficacy requires self-control (DeBono and Muraven, 2013). Workers' self-control resources reduced under the state of ego depletion, thus leading to decreased self-efficacy. Given this, we proposed that there existed multiple-step mediating effects through ego depletion and self-efficacy. However, the empirical results only supported part of our hypothesis about the continuous mediating effects. Only the multiple-step mediating effect of safety role conflict → ego depletion → self-efficacy → safety participation and the multiple-step of interpersonal safety conflict → ego depletion → self-efficacy → safety compliance were significant. It meant that safety role conflict may influence workers' safety participation through the multiple-step mediation of ego depletion and self-efficacy. And interpersonal safety conflict may affect workers' safety compliance through the multiple-step mediation of ego depletion and self-efficacy. Although only part of the continuous mediating effects was supported, it was still a useful attempt to explore the complex mechanism between safety stressors and safety performance.

### Practical Implications

The results of this study also bring a lot of practical implications. First, in view of the negative effects of safety stressors on safety performance, construction enterprises should systematically identify safety stressors that workers may experience and take measures to eliminate them. Clear job description, safety information and safety role expectation should be provided to workers to avoid safety role ambiguity. Project managers should organize unified training for personnel from different organizations (e.g., sub-contractors) to form consistent safety cognition, thus reducing safety role conflict. In the meanwhile, cooperation should be emphasized to avoid interpersonal safety conflict. However, due to the unique characteristics of the construction industry, safety stressors may not be completely eradicated. Therefore, construction managers should also pay attention to regulating workers' psychological and physiological states. Workers should be provided with more chances to improve their safety knowledge and skills to improve their self-efficacy. Construction enterprises should also develop more reasonable work schedules to ensure that workers have enough rest time to mitigate ego depletion.

### Limitations and Future Research

Limitations and suggestions for future research are as follows. First, this study measured safety stressors, ego depletion, self-efficacy, and safety performance with cross-sectional data. The cross-sectional data is not conducive to revealing the dynamic process in which safety stressors affect safety performance through ego depletion and self-efficacy. Longitudinal data are suggested for future studies. Second, the generalizability of results in this study might be limited because the sample data were obtained from construction workers in western China. Future studies may consider extending the sample to more regions and other high-risk industries. Third, this study only considered three safety stressors, and workers may face other safety stressors as well. As the relationship between job stressors and job performance changes with the type of stressors tested, future studies can explore more types of safety stressors and compare their effects. Finally, ego depletion only mediated part of the relationships between safety stressors and safety performance, and only part of the multiple-step mediating effects through ego depletion and self-efficacy were supported, suggesting that more examination should be conducted in the future.

## Conclusion

This study investigated the effects of three types of safety stressors on construction workers' safety performance and the potential mediating role of ego depletion and self-efficacy in the relationship between these safety stressors and safety performance. The results showed that safety role ambiguity, safety role conflict and interpersonal safety conflict negatively affected workers' safety compliance and safety participation. Ego depletion was found to mediate the relationship between interpersonal safety conflict and safety compliance, the relationship between safety role conflict and safety participation, as well as the relationship between interpersonal safety conflict and safety participation. Self-efficacy mediated all the examined relationships between the three types of safety stressors and workers' safety performance. Additionally, we found that safety role conflict may influence workers' safety participation through the multiple-step mediation of ego depletion and self-efficacy, and interpersonal safety conflict may affect workers' safety compliance through the multiple-step mediation of ego depletion and self-efficacy. These findings help to clarify how safety stressors influence workers' safety performance and expand the scope of application of the ego depletion theory, job demands-resources model, and conservation of resources theory. This study also makes contributions by providing more empirical evidence for the relationship between safety stressors and safety performance. Moreover, this study has proposed many practical suggestions for improving workers' safety performance.

## Data Availability Statement

The original contributions presented in the study are included in the article/supplementary material, further inquiries can be directed to the corresponding authors.

## Author Contributions

GY: conceptualization, supervision, funding acquisition, and resources. QX: writing—original draft, writing—review and editing, formal analysis, and validation. LY: investigation, methodology, software, and visualization. JY, NX, YL, and TH: writing—review and editing. All authors contributed to the article and approved the submitted version.

## Funding

This work was supported by the National Natural Science Foundation of China [grant number 71972020]; the Fundamental Research Funds for the Central Universities [grant number 2020CDJK03ZH04]; and the Graduate Scientific Research and Innovation Foundation of Chongqing [grant number CYB20040].

## Conflict of Interest

The authors declare that the research was conducted in the absence of any commercial or financial relationships that could be construed as a potential conflict of interest.

## Publisher's Note

All claims expressed in this article are solely those of the authors and do not necessarily represent those of their affiliated organizations, or those of the publisher, the editors and the reviewers. Any product that may be evaluated in this article, or claim that may be made by its manufacturer, is not guaranteed or endorsed by the publisher.

## References

[B1] Abbas M. Raja U. (2019). Challenge-hindrance stressors and job outcomes: the moderating role of conscientiousness. J. Bus. Psychol. 34, 189–201. 10.1007/s10869-018-9535-z

[B2] Adjekum D. K. (2017). An evaluation of the relationships between collegiate aviation safety management system initiative, self-efficacy, transformational safety leadership and safety behavior mediated by safety motivation. Int. J. Aviat. Aeronaut. Aerospace 4:1169. 10.15394/ijaa.2017.1169

[B3] Akgunduz Y. (2015). The influence of self-esteem and role stress on job performance in hotel businesses. Int. J. Contemp. Hospital. Manag. 27, 1082–1099. 10.1108/IJCHM-09-2013-0421

[B4] Al-Bsheish M. A. Mustafa M. B. Ismail M. A. (2017). Enhancing safety performance by recognizing the role of perceived management commitment to safety in the jordanian healthcare industry: conceptual framework. Int. J. Bus. Soc. Res. 7:1023. 10.18533/ijbsr.v7i01.1023

[B5] Al-Kasasbeh M. Abudayyeh O. Olimat H. Liu H. X. Al Mamlook R. Alfoul B. A. (2021). A robust construction safety performance evaluation framework for workers' compensation insurance: a proposed alternative to EMR. Buildings 11:434. 10.3390/buildings11100434

[B6] Alroomi A. S. Mohamed S. (2021). Occupational stressors and safety behaviour among oil and gas workers in Kuwait: the mediating role of mental health and fatigue. Int. J. Environ. Res. Public Health 18:2111700. 10.3390/ijerph182111700 34770215 PMC8583007

[B7] Ashill N. J. Rod M. (2011). Burnout processes in non-clinical health service encounters. J. Bus. Res. 64, 1116–1127. 10.1016/j.jbusres.2010.11.004

[B8] Bakker A. B. Demerouti E. Euwema M. C. (2005). Job resources buffer the impact of job demands on burnout. J. Occup. Health Psychol. 10, 170–180. 10.1037/1076-8998.10.2.170 15826226

[B9] Bandura A. (1977). Self-efficacy: toward a unifying theory of behavioral change. Adv. Behav. Res. Ther. 84, 139–161. 10.1037/0033-295X.84.2.191 847061

[B10] Barki H. Hartwick J. (2004). Conceptualizing the construct of interpersonal conflict. Int. J. Confl. Manag. 15, 216–244. 10.1108/eb022913

[B11] Barnes C. M. Wagner D. T. (2009). Changing to daylight saving time cuts into sleep and increases workplace injuries. J. Appl. Psychol. 94, 1305–1317. 10.1037/a0015320 19702372

[B12] Baron R. M. Kenny D. A. (1986). The moderator-mediator variable distinction in social psychological research. Conceptual, strategic, and statistical considerations. J. Personal. Soc. Psychol. 51, 1173–1182. 10.1037/0022-3514.51.6.1173 3806354

[B13] Baumeister R. F. Bratslavsky E. Muraven M. Tice D. M. (1998). Ego depletion: is the active self a limited resource? J. Personal. Soc. Psychol. 74, 1252–1265. 10.1037/0022-3514.74.5.1252 9599441

[B14] Baumeister R. F. Vohs K. D. Tice D. M. (2007). The strength model of self-control. Curr. Direct. Psychol. Sci. 16, 351–355. 10.1111/j.1467-8721.2007.00534.x

[B15] Beehr T. A. Bhagat R. S. (1985). Human Stress and Cognition in Organizations: An Integrated Perspective. New York, NY: Wiley.

[B16] Bertrams A. Pahl S. (2014). Ego depletion after social interference. Psychology 5, 1–5. 10.4236/psych.2014.51001

[B17] Borman W. C. Motowidlo S. J. (1993). Expanding the criterion domain to include elements of contextual performance, in Personnel Selection in Organizations. eds Schmitt N. Borman, Associates W. C. (San Francisco: Jossey-Bass), 71–98.

[B18] Brockman J. L. (2014). Interpersonal conflict in construction: cost, cause, and consequence. J. Constr. Eng. Manag. 140:805. 10.1061/(ASCE)CO.1943-7862.0000805

[B19] Cavanaugh M. A. Boswell W. R. Roehling M. V. Boudreau J. W. (2000). An empirical examination of self-reported work stress among U.S. managers. J. Appl. Psychol. 85:65. 10.1037/0021-9010.85.1.65 10740957

[B20] Celik K. (2013). The effect of role ambiguity and role conflict on performance of vice principals: the mediating role of burnout. Eur. J. Educ. Res. 13, 195–214. 10.1002/chp.21176 23775915

[B21] Che X. X. Zhou Z. Q. E. Kessler S. R. Spector P. E. (2017). Stressors beget stressors: the effect of passive leadership on employee health through workload and work-family conflict. Work Stress 31, 338–354. 10.1080/02678373.2017.1317881

[B22] Chebat J. C. Kollias P. (2000). The impact of empowerment on customer contact employees' roles in service organizations. J. Serv. Res. 3, 66–81. 10.1177/109467050031005

[B23] Chen C. F. Chen S. C. (2014). Measuring the effects of Safety Management System practices, morality leadership and self-efficacy on pilots' safety behaviors: Safety motivation as a mediator. Saf. Sci. 62, 376–385. 10.1016/j.ssci.2013.09.013

[B24] Cheung G. W. Lau R. S. (2008). Testing mediation and suppression effects of latent variables: bootstrapping with structural equation models. Org. Res. Method. 11, 296–325. 10.1177/1094428107300343

[B25] Chow J. T. Hui C. M. Lau S. (2015). A depleted mind feels inefficacious: ego-depletion reduces self-efficacy to exert further self-control. Eur. J. Soc. Psychol. 45, 754–768. 10.1002/ejsp.212025855820

[B26] Christian M. S. Ellis A. P. J. (2011). Examining the effects of sleep deprivation on workplace deviance: a self-regulatory perspective. Acad. Manag. J. 54, 913–934. 10.5465/amj.2010.0179

[B27] Chughtai A. A. (2015). Creating safer workplaces: the role of ethical leadership. Saf. Sci. 73, 92–98. 10.1016/j.ssci.2014.11.016

[B28] Curcuruto M. Conchie S. M. Griffin M. A. (2019). Safety citizenship behavior (SCB) in the workplace: a stable construct? Analysis of psychometric invariance across four European countries. Accid. Anal. Prev. 129, 190–201. 10.1016/j.aap.2019.05.023 31163325

[B29] Dai H. Milkman K. L. Hofmann D. A. Staats B. R. (2015). The impact of time at work and time off from work on rule compliance: the case of hand hygiene in health care. J. Appl. Psychol. 100, 846–862. 10.1037/a0038067 25365728

[B30] De Jonge J. Dormann C. (2006). Stressors, resources, and strain at work: a longitudinal test of the triple-match principle. J. Appl. Psychol. 91, 1359–1374. 10.1037/0021-9010.91.5.1359 17100490

[B31] De Silva N. Samanmali R. De Silva H. L. (2017). Managing occupational stress of professionals in large construction projects. J. Eng. Des. Technol. 15, 488–504. 10.1108/JEDT-09-2016-0066

[B32] DeBono A. Muraven M. (2013). Keeping it real: self-control depletion increases accuracy, but decreases confidence for performance. J. Appl. Soc. Psychol. 43, 879–886. 10.1111/jasp.12013

[B33] Demerouti E. Bakker A. B. Nachreiner F. Schaufeli W. B. (2001). The job demands-resources model of burnout. J. Appl. Psychol. 86, 499–512. 10.1037/0021-9010.86.3.499 11419809

[B34] Dewall C. N. Baumeister R. F. Stillman T. F. Gailliot M. T. (2007). Violence restrained: effects of self-regulation and its depletion on aggression. J. Exp. Soc. Psychol. 43, 62–76. 10.1016/j.jesp.2005.12.005

[B35] Diestel S. Schmidt K. H. (2011). Costs of simultaneous coping with emotional dissonance and self-control demands at work: results from two German samples. J. Appl. Psychol. 96:643. 10.1037/a0022134 21142345

[B36] Dzeng R. J. Lin C. T. Fang Y. C. (2016). Using eye-tracker to compare search patterns between experienced and novice workers for site hazard identification. Saf. Sci. 82, 56–67. 10.1016/j.ssci.2015.08.008

[B37] Eatough E. M. Chang C. H. Miloslavic S. A. Johnson R. E. (2011). Relationships of role stressors with organizational citizenship behavior: a meta-analysis. J. Appl. Psychol. 96, 619–632. 10.1037/a0021887 21244128

[B38] Eys M. A. Carron A. V. (2001). Role ambiguity, task cohesion, and task self-efficacy. Small Gr. Res. 32, 356–373. 10.1177/104649640103200305

[B39] Fang D. P. Huang Y. C. Guo H. L. Lim H. W. (2020). LCB approach for construction safety. Saf. Sci. 128:104761. 10.1016/j.ssci.2020.104761

[B40] Fang D. P. Zhao C. Zhang M. C. (2016). A cognitive model of construction workers' unsafe behaviors. J. Constr. Eng. Manag. 142:1118. 10.1061/(ASCE)CO.1943-7862.0001118

[B41] Fischer P. Greitemeyer T. Frey D. (2007). Ego depletion and positive illusions: does the construction of positivity require regulatory resources? Personal. Soc. Psychol. Bullet. 33, 1306–1321. 10.1177/0146167207303025 17578935

[B42] Fornell C. Larcker D. F. (1981). Structural equation models with unobservable variables and measurement error: algebra and statistics. J. Market. Res. 18, 382–388. 10.1177/002224378101800313

[B43] Gittleman J. L. Gardner P. C. Haile E. Sampson J. M. Cigularov K. P. Ermann E. D.. (2010). Case Study CityCenter and Cosmopolitan Construction Projects, Las Vegas, Nevada: lessons learned from the use of multiple sources and mixed methods in a safety needs assessment. J. Saf. Res. 41, 263–281. 10.1016/j.jsr.2010.04.004 20630278

[B44] Grabowski M. Ayyalasomayajula P. Merrick J. Harrald J. R. Roberts K. (2007). Leading indicators of safety in virtual organizations. Saf. Sci. 45, 1013–1043. 10.1016/j.ssci.2006.09.007

[B45] Graham J. D. Ginis K. A. M. Bray S. R. (2017). Exertion of self-control increases fatigue, reduces task self-efficacy, and impairs performance of resistance exercise. Sport Exerc. Performance Psychol. 6, 70–88. 10.1037/spy0000074

[B46] Graham J. D. Steven B. (2015). Self-control strength depletion reduces self-efficacy to exert self-control, task self-efficacy, and impairs resistance exercise performance. J. Sport Exerc. Psychol. 37:477. 10.1123/jsep.2015-0064 26524094

[B47] Grebner S. Elfering A. Semmer N. K. (2010). The Success Resource Model of Job Stress. Bingley: Emerald. 10.1108/S1479-3555(2010)0000008005

[B48] Griffin M. A. Hu X. (2013). How leaders differentially motivate safety compliance and safety participation: the role of monitoring, inspiring, and learning. Saf. Sci. 60, 196–202. 10.1016/j.ssci.2013.07.019

[B49] Griffin M. A. Neal A. (2000). Perceptions of safety at work: a framework for linking safety climate to safety performance, knowledge, and motivation. J. Occup. Health Psychol. 5, 347–358. 10.1037/1076-8998.5.3.347 10912498

[B50] Guarnaccia C. Scrima F. Civilleri A. Salerno L. (2018). The role of occupational self-efficacy in mediating the effect of job insecurity on work engagement, satisfaction and general health. Curr. Psychol. 37, 488–497. 10.1007/s12144-016-9525-0

[B51] Gunduz M. Birgonul M. T. Ozdemir M. (2018). Development of of a safety performance index assessment tool by using a fuzzy structural equation model for construction sites. Automat. Constr. 85, 124–134. 10.1016/j.autcon.2017.10.012

[B52] Hagger M. S. Wood C. Stiff C. Chatzisarantis N. L. D. (2010). Ego depletion and the strength model of self-control: a meta-analysis. Psychol. Bullet. 136, 495–525. 10.1037/a0019486 20565167

[B53] Hair J. F. Black W. C. Babin B. J. Anderson R. E. Tatham R. L. (2006). Multivariate Data Analysis, 6th Edn. Hoboken, NJ: Prentice-Hall.

[B54] Halbesleben J. R. B. Neveu J.-P. Paustian-Underdahl S. C. Westman M. (2014). Getting to the “COR”: understanding the role of resources in conservation of resources theory. J. Manag. 40, 1334–1364. 10.1177/0149206314527130

[B55] Hale A. Borys D. (2013). Working to rule, or working safely? Part 1: a state of the art review. Saf. Sci. 55, 207–221. 10.1016/j.ssci.2012.05.011

[B56] Hartline M. D. Ferrell O. C. (1996). The management of customer-contact service employees: an empirical investigation. J. Market. 60, 52–70. 10.1177/002224299606000406

[B57] Hasanzadeh S. Dao B. Esmaeili B. Dodd M. D. (2019). Role of personality in construction safety: investigating the relationships between personality, attentional failure, and hazard identification under fall-hazard conditions. J. Constr. Eng. Manag. 145:1673. 10.1061/(ASCE)CO.1943-7862.0001673

[B58] He C. McCabe B. Jia G. Sun J. (2020). Effects of safety climate and safety behavior on safety outcomes between supervisors and construction workers. J. Constr. Eng. Manag. 146:1735. 10.1061/(ASCE)CO.1943-7862.000173529776527

[B59] He C. Q. Jia G. S. McCabe B. Chen Y. T. Sun J. D. (2019). Impact of psychological capital on construction worker safety behavior: communication competence as a mediator. J. Saf. Res. 71, 231–241. 10.1016/j.jsr.2019.09.007 31862034

[B60] Hinsz V. B. Nickell G. S. Park E. S. (2007). The role of work habits in the motivation of food safety behaviors. J. Exp. Psychol. Appl. 13, 105–114. 10.1037/1076-898X.13.2.105 17535135

[B61] Hinze J. Thurman S. Wehle A. (2013). Leading indicators of construction safety performance. Saf. Sci. 51, 23–28. 10.1016/j.ssci.2012.05.016

[B62] Hobfoll S. E. (1989). Conservation of resources - a new attempt at conceptualizing stress. Am. Psychol. 44, 513–524. 10.1037/0003-066X.44.3.513 2648906

[B63] Hobfoll S. E. Halbesleben J. Neveu J.-P. Westman M. (2018). Conservation of resources in the organizational context: the reality of resources and their consequences. Ann. Rev. Org. Psychol. Org. Behav. 5, 103–128. 10.1146/annurev-orgpsych-032117-104640

[B64] Jackson S. E. Schuler R. S. (1985). A meta-analysis and conceptual critique of research on role ambiguity and role conflict in work settings. Org. Behav. Hum. Decision Proces. 36, 16–78. 10.1016/0749-5978(85)90020-2

[B65] Jex S. M. (1998). Stress and Job Performance: Theory, Research, and Implications for Managerial Practice. Thousand Oaks, CA: Sage Publications.

[B66] Jex S. M. Gudanowski D. M. (1992). Efficacy beliefs and work stress - an exploratory-study. J. Org. Behav. 13, 509–517. 10.1002/job.403013050618825567

[B67] Jiang J. Y. Zhang X. Tjosvold D. (2013). Emotion regulation as a boundary condition of the relationship between team conflict and performance: a multi-level examination. J. Org. Behav. 34, 714–734. 10.1002/job.183425855820

[B68] Jiang Z. M. Fang D. P. Zhang M. C. (2015). Understanding the causation of construction workers' unsafe behaviors based on system dynamics modeling. J. Manag. Eng. 31:350. 10.1061/(ASCE)ME.1943-5479.0000350

[B69] Job V. Dweck C. S. Walton G. M. (2010). Ego depletion-is it all in your head? Implicit theories about willpower affect self-regulation. Psychol. Sci. 21, 1686–1693. 10.1177/0956797610384745 20876879

[B70] Johnson R. E. Lanaj K. Barnes C. M. (2014). The good and bad of being fair: effects of procedural and interpersonal justice behaviors on regulatory resources. J. Appl. Psychol. 99, 635–650. 10.1037/a0035647 24446913

[B71] Kadir A. R. Kamariah N. Saleh A. Ratnawati A. (2017). The effect of role stress, job satisfaction, self-efficacy and nurses' adaptability on service quality in public hospitals of Wajo. Int. J. Qual. Serv. Sci. 9, 184–202. 10.1108/IJQSS-10-2016-0074

[B72] Karatepe O. M. Yavas U. Babakus E. Avci T. (2006). Does gender moderate the effects of role stress in frontline service jobs? J. Bus. Res. 59, 1087–1093. 10.1016/j.jbusres.2006.08.004

[B73] Kilroy S. Flood P. C. Bosak J. Chenevert D. (2016). Perceptions of high-involvement work practices and burnout: the mediating role of job demands. Hum. Resour. Manag. J. 26, 408–424. 10.1111/1748-8583.12112

[B74] Kim B. J. Jung S. Y. (2019). The mediating role of job strain in the transformational leadership-safety behavior link: the buffering effect of self-efficacy on safety. Int. J. Environ. Res. Public Health 16:81425. 10.3390/ijerph16081425 31010078 PMC6518062

[B75] Kim M. Beehr T. A. (2018). Challenge and hindrance demands lead to employees' health and behaviours through intrinsic motivation. Stress Health 34, 367–378. 10.1002/smi.2796 29327495

[B76] Kim N. Kang Y. J. Choi J. Sohn Y. W. (2020). The crossover effects of supervisors' workaholism on subordinates' turnover intention: the mediating role of two types of job demands and emotional exhaustion. Int. J. Environ. Res. Public Health 17:217742. 10.3390/ijerph17217742 33113900 PMC7660161

[B77] Lepine J. A. Podsakoff N. P. Lepine M. A. (2005). A meta-analytic test of the challenge stressor-hindrance stressor framework: an explanation for inconsistent relationships among stressors and performance. Acad. Manag. J. 48, 764–775. 10.5465/amj.2005.18803921

[B78] Leung M.-y. Liang Q. Chan I. Y. S. (2017). Development of a stressors-stress-performance-outcome model for expatriate construction professionals. J. Constr. Eng. Manag. 143:1266. 10.1061/(ASCE)CO.1943-7862.000126626643649

[B79] Leung M. Y. Liang Q. Olomolaiye P. (2016). Impact of job stressors and stress on the safety behavior and accidents of construction workers. J. Manag. Eng. 32:373. 10.1061/(ASCE)ME.1943-5479.0000373

[B80] Li A. Bagger J. (2008). Role ambiguity and self-efficacy: the moderating effects of goal orientation and procedural justice. J. Voc. Behav. 73, 368–375. 10.1016/j.jvb.2008.07.008

[B81] Li H. Lu M. Hsu S.-C. Gray M. Huang T. (2015). Proactive behavior-based safety management for construction safety improvement. Saf. Sci. 75, 107–117. 10.1016/j.ssci.2015.01.013

[B82] Liang Q. Leung M.-, y. Ahmed K. (2021). How adoption of coping behaviors determines construction workers' safety: a quantitative and qualitative investigation. Saf. Sci. 133:105035. 10.1016/j.ssci.2020.105035

[B83] Lin S. H. Ma J. Johnson R. E. (2016). When ethical leader behavior breaks bad: how ethical leader behavior can turn abusive *via* ego depletion and moral licensing. J. Appl. Psychol. 101, 815–830. 10.1037/apl0000098 26867103

[B84] Lorente Prieto L. Salanova Soria M. Martinez Martinez I. Schaufeli W. (2008). Extension of the Job Demands-Resources model in the prediction of burnout and engagement among teachers over time. Psicothema 20, 354–360. 10.1037/0735-7028.39.4.471 18674427

[B85] Lu C. Q. Du D. Y. Xu X. M. (2016). What differentiates employees' job performance under stressful situations: the role of general self-efficacy. J. Psychol. 150, 837–848. 10.1080/00223980.2016.1203277 27419467

[B86] Martinez-Corts I. Demerouti E. Bakker A. B. Boz M. (2015). Spillover of interpersonal conflicts from work into nonwork: a daily diary study. J. Occup. Health Psychol. 20, 326–337. 10.1037/a0038661 25602278

[B87] Maslach C. (1982). Understanding Burnout: Definition Issues in Analyzing a Complex Phenomenon. Beverly Hills, CA: Sage.

[B88] Melia J. L. Becerril M. (2007). Psychosocial sources of stress and burnout in the construction sector: a structural equation model. Psicothema 19, 679–686. 10.1016/j.nlm.2007.06.001 17959126

[B89] Ministry of Emergency Management of the People's Republic of China (2018). Circular of the State Council's Office of the Security Council on the State of Work Safety in the Construction Industry in the First Half of 2018. Available online at: https://www.mem.gov.cn/gk/tzgg/tb/201807/t20180725_230568.shtml

[B90] Mohr G. Wolfram H.-J. (2010). Stress among managers: the importance of dynamic tasks, predictability, and social support in unpredictable times. J. Occup. Health Psychol. 15, 167–179. 10.1037/a0018892 20364914

[B91] Moosa M. H. Oriet L. P. (2021). Factors affecting safety performance in the construction industry: an empirical study using structural equation modelling. Int. J. Occup. Saf. Ergon. 2021, 1–11. 10.1080/10803548.2021.1985302 34704541

[B92] Muraven M. Baumeister R. F. (2000). Self-regulation and depletion of limited resources: does self-control resemble a muscle? Psychol. Bullet. 126, 247–259. 10.1037/0033-2909.126.2.247 10748642

[B93] Neal A. Griffin M. A. (1997). Perceptions of Safety at Work: Developing a Model to Link Organizational Safety Climate and Individual Behavior, in 12th Annual Conference of the Society for Industrial and Organizational Psychology. St. Louis, MO.

[B94] Nodoushan R. J. Akhavan A. Miyanshahri M. E. Anoosheh V. S. (2020). Investigation of the relationship between occupational cognitive failures and work-related accidents in heavy equipment operators of Shahid Rajaee port complex. J. Educ. Health Promot. 9:19. 10.4103/jehp.jehp_577_19 32953915 PMC7482705

[B95] Nunnally J. C. (1978). Psychometric Theory. New York, NY: McGraw-Hill.

[B96] Occupational Safety Health Administration (2019). Fatal Occupational Injuries Counts and Rates by Selected Industries. Available online at: https://www.bls.gov/news.release/cfoi.t04.htm

[B97] Parker S. K. (2000). From passive to proactive motivation: the importance of flexible role orientations and role breadth self-efficacy. Appl. Psychol. 49, 447–469. 10.1111/1464-0597.00025

[B98] Prapanjaroensin A. Patrician P. A. Vance D. E. (2017). Conservation of resources theory in nurse burnout and patient safety. J. Adv. Nurs. 73, 2558–2565. 10.1111/jan.13348 28543427

[B99] Prem R. Kubicek B. Diestel S. Korunka C. (2016). Regulatory job stress ors and their within-person relationships with ego depletion: the roles of state anxiety, self-control effort, and job autonomy. J. Voc. Behav. 92, 22–32. 10.1016/j.jvb.2015.11.004

[B100] Probst T. M. Brubaker T. L. (2001). The effects of job insecurity on employee safety outcomes: cross-sectional and longitudinal explorations. J. Occup. Health Psychol. 6, 139–159. 10.1037/1076-8998.6.2.139 11326726

[B101] Qi H. N. Zhou Z. P. Li N. Zhang C. G. (2022). Construction safety performance evaluation based on data envelopment analysis (DEA) from a hybrid perspective of cross-sectional and longitudinal. Saf. Sci. 146:105532. 10.1016/j.ssci.2021.105532

[B102] Reason J. Parker D. Lawton R. (1998). Organizational controls and safety: the varieties of rule-related behaviour. J. Occup. Org. Psychol. 71, 289–304. 10.1111/j.2044-8325.1998.tb00678.x

[B103] Righetti F. Finkenauer C. (2011). If you are able to control yourself, i will trust you: the role of perceived self-control in interpersonal trust. J. Personal. Soc. Psychol. 100, 874–886. 10.1037/a0021827 21319912

[B104] Rizzo J. R. House R. J. Lirtzman S. I. (1970). Role conflict and ambiguity in complex organizations. Admin. Sci. Quart. 15, 150–163. 10.2307/2391486847478

[B105] Rosen C. Chang C.-H. Djurdjevic E. Eatough E. (2010). Occupational stressors and job performance: an updated review and recommendations, in: New Developments in Theoretical and Conceptual Approaches to Job Stress, eds Perrewe P. L. Ganster D. C. (Bradford: Emerald Group Publishing Limited), 1–60. 10.1108/S1479-3555(2010)0000008004

[B106] Salmon S. J. Fennis B. M. Ridder D. T. D. De Adriaanse M. A. Emely D. V. (2014). Health on impulse: when low self-control promotes healthy food choices. Health Psychol. 33, 103–109. 10.1037/a0031785 23477580

[B107] Sampson J. M. DeArmond S. Chen P. Y. (2014). Role of safety stressors and social support on safety performance. Saf. Sci. 64, 137–145. 10.1016/j.ssci.2013.11.025

[B108] Sanni-Anibire M. O. Mahmoud A. S. Hassanain M. A. Salami B. A. (2020). A risk assessment approach for enhancing construction safety performance. Saf. Sci. 121, 15–29. 10.1016/j.ssci.2019.08.044

[B109] Schaufeli W. B. (2017). Applying the job demands-resources model: a 'how to' guide to measuring and tackling work engagement and burnout. Org. Dyn. 46, 120–132. 10.1016/j.orgdyn.2017.04.008

[B110] Schaufeli W. B. Bakker A. B. (2004). Job demands, job resources, and their relationship with burnout and engagement: a multi-sample study. J. Org. Behav. 25, 293–315. 10.1002/job.24825855820

[B111] Schmeichel B. J. Vohs K. D. Baumeister R. F. (2003). Intellectual performance and ego depletion: role of the self in logical reasoning and other information processing. J. Personal. Soc. Psychol. 85, 33–46. 10.1037/0022-3514.85.1.33 12872883

[B112] Schmidt K.-H. Neubach B. Heuer H. (2007). Self-control demands, cognitive control deficits, and burnout. Work Stress 21, 142–154. 10.1080/02678370701431680

[B113] Schwarzer R. Bäßler J. Kwiatek P. Schröder K. Zhang J. X. (1997). The assessment of optimistic self-beliefs: comparison of the German, Spanish, and Chinese versions of the general self-efficacy scale. Appl. Psychol. 46, 69–88. 10.1111/j.1464-0597.1997.tb01096.x

[B114] Seo D. C. (2005). An explicative model of unsafe work behavior. Saf. Sci. 43, 187–211. 10.1016/j.ssci.2005.05.001

[B115] Shaikh A. Y. Osei-Kyei R. Hardie M. (2021). A critical analysis of safety performance indicators in construction. Int. J. Build. Pathol. Adapt. 39, 547–580. 10.1108/IJBPA-03-2020-0018

[B116] Sinelnikov S. Inouye J. Kerper S. (2015). Using leading indicators to measure occupational health and safety performance. Saf. Sci. 72, 240–248. 10.1016/j.ssci.2014.09.01011843425

[B117] Sonnentag S. Jelden S. (2009). Job stressors and the pursuit of sport activities: a day-level perspective. J. Occup. Health Psychol. 14, 165–181. 10.1037/a0014953 19331478

[B118] Spector P. E. Jex S. M. (1998). Development of four self-report measures of job stressors and strain: Interpersonal Conflict at Work Scale, Organizational Constraints Scale, Quantitative Workload Inventory, and Physical Symptoms Inventory. J. Occup. Health Psychol. 3, 356–367. 10.1037/1076-8998.3.4.356 9805281

[B119] Tang Y.-T. Chang C.-H. (2010). Impact of role ambiguity and role conflict on employee creativity. Afr. J. Bus. Manag. 4, 869–881. 10.5897/AJBM.9000334

[B120] Tangney J. P. Baumeister R. F. Boone A. L. (2004). High self-control predicts good adjustment, less pathology, better grades, and interpersonal success. J. Perfsonal. 72, 271–324. 10.1111/j.0022-3506.2004.00263.x 15016066

[B121] Ten Brummelhuis L. L. Haar J. M. Roche M. (2014). Does family life help to be a better leader? a closer look at crossover processes from leaders to followers. Person. Psychol. 67, 917–949. 10.1111/peps.12057

[B122] Toellner J. (2001). Improving safety and health performance: identifying and measuring leading indicators. Profession. Saf. 46:42. 10.1021/ja8034027 18774821

[B123] Trougakos J. P. Beal D. J. Cheng B. H. Hideg I. Zweig D. (2015). Too drained to help: a resource depletion perspective on daily interpersonal citizenship behaviors. J. Appl. Psychol. 100:227. 10.1037/a0038082 25314365

[B124] Wallace J. C. Arnold T. Finch D. M. Edwards B. D. Frazier M. L. (2009). Work stressors, role-based performance, and the moderating influence of organizational support. J. Appl. Psychol. 94, 254–262. 10.1037/a0013090 19186910

[B125] Wang D. Wang X. Griffin M. A. Wang Z. (2020). Safety stressors, safety-specific trust, and safety citizenship behavior: a contingency perspective. Accid. Anal. Prev. 142:105572. 10.1016/j.aap.2020.105572 32361476

[B126] Wang D. Wang X. Xia N. (2018). How safety-related stress affects workers' safety behavior: the moderating role of psychological capital. Saf. Sci. 103, 247–259. 10.1016/j.ssci.2017.11.020

[B127] Wu G. D. Hu Z. B. Zheng J. W. (2019). Role stress, job burnout, and job performance in construction project managers: the moderating role of career calling. Int. J. Environ. Res. Public Health 16:132394. 10.3390/ijerph16132394 31284496 PMC6651169

[B128] Xia A. Wang B. Song B. Zhang W. Qian J. (2019). How and when workplace ostracism influences task performance: through the lens of conservation of resource theory. Hum. Resour. Manag. J. 29, 353–370. 10.1111/1748-8583.12226

[B129] Xia N. N. Xie Q. H. Hu X. W. Wang X. Q. Meng H. (2020a). A dual perspective on risk perception and its effect on safety behavior: a moderated mediation model of safety motivation, and supervisor's and coworkers' safety climate. Accid. Anal. Prev. 134:105350. 10.1016/j.aap.2019.105350 31715549

[B130] Xia Y. Schyns B. Zhang L. (2020b). Why and when job stressors impact voice behaviour: an ego depletion perspective. J. Bus. Res. 109, 200–209. 10.1016/j.jbusres.2019.11.053

[B131] Zhang R. P. Lingard H. Oswald D. (2020). Impact of supervisory safety communication on safety climate and behavior in construction workgroups. J. Constr. Eng. Manag. 146:1881. 10.1061/(ASCE)CO.1943-7862.0001881

[B132] Zhong L. Qian Z. Wang D. (2020). How does the servant supervisor influence the employability of postgraduates? Exploring the mechanisms of self-efficacy and academic engagement. Front. Bus. Res. China 14:1. 10.1186/s11782-020-00079-1

[B133] Zhou Z. Irizarry J. Guo W. (2021). A network-based approach to modeling safety accidents and causations within the context of subway construction project management. Saf. Sci. 139:105261. 10.1016/j.ssci.2021.105261

